# A stochastic model for circadian rhythms from coupled ultradian oscillators

**DOI:** 10.1186/1742-4682-4-1

**Published:** 2007-01-09

**Authors:** Roderick Edwards, Richard Gibson, Reinhard Illner, Verner Paetkau

**Affiliations:** 1 Department of Mathematics and Statistics, University of Victoria, P.O. Box 3045 STN CSC, Victoria, BC, V8W 3P4, Canada; 2 Department of Biochemistry and Microbiology, University of Victoria, P.O. Box 3055 STN CSC, Victoria, BC, V8W 3P6, Canada

## Abstract

**Background:**

Circadian rhythms with varying components exist in organisms ranging from humans to cyanobacteria. A simple evolutionarily plausible mechanism for the origin of such a variety of circadian oscillators, proposed in earlier work, involves the non-disruptive coupling of pre-existing ultradian transcriptional-translational oscillators (TTOs), producing "beats," in individual cells. However, like other TTO models of circadian rhythms, it is important to establish that the inherent stochasticity of the protein binding and unbinding does not invalidate the finding of clear oscillations with circadian period.

**Results:**

The TTOs of our model are described in two versions: 1) a version in which the activation or inhibition of genes is regulated stochastically, where the 'unoccupied" (or "free") time of the site under consideration depends on the concentration of a protein complex produced by another site, and 2) a deterministic, "time-averaged" version in which the switching between the "free" and "occupied" states of the sites occurs so rapidly that the stochastic effects average out. The second case is proved to emerge from the first in a mathematically rigorous way. Numerical results for both scenarios are presented and compared.

**Conclusion:**

Our model proves to be robust to the stochasticity of protein binding/unbinding at experimentally determined rates and even at rates several orders of magnitude slower. We have not only confirmed this by numerical simulation, but have shown in a mathematically rigorous way that the time-averaged deterministic system is indeed the fast-binding-rate limit of the full stochastic model.

## Background

We are concerned with mechanisms that can account for circadian rhythms at the cellular level. Although circadian oscillators exist in complex multicellular organisms as well as in single-cell organisms, it is thought that most occur in single cells [1-3]. We have previously [4] described a model for circadian oscillations in which ultradian oscillators, which have been widely observed to occur in living systems, are coupled to produce circadian periods. The model was based, as is much of the related literature, on so-called transcriptional-translational oscillators (TTOs), in which genes are activated or inhibited for transcription by protein products of the oscillating system itself (transcriptional activators or suppressors, respectively). Several models for interactions between more than one oscillator to generate a circadian one have been described [5-7], but ours differs in positing coupling between the protein products of *independent *ultradian oscillators. We argued that our model provides a plausible evolutionary origin for circadian oscillators across a range of organisms, since it allows existing ultradian oscillations to be co-opted as components of circadian oscillators without disturbing their primary functions.

A challenging feature of TTOs is the fact that in cells, a given transcribed gene is present in one, or at most a small number, of copies, and its interaction with a transcriptional regulator is not correctly modeled by deterministic differential equations as used in [4]. Rather, because the number of copies of an expressed gene, and at some times the numbers of transcription factor molecules in a cell is small, such interactions are more accurately described by stochastic equations, and this has been done for a number of existing models [6,8-10], using a classical algorithm due to Gillespie [11]. In some cases this results in shorter autocorrelation times [8] or random fluctuations [6]. Typically, as for example in reference [9], the effect of the stochasticity is to degrate the circadian oscillations, but for fast enough binding rates, the circadian oscillations are maintained. Our objective here is to apply the stochastic approach to a model similar to the one described in [4]. To do this, we have estimated the rates of association and dissociation of transcription factors from their DNA binding sites. We have then incorporated these rates, together with parameters previously used [4], into the new version of the model, in which the DNA binding steps have been treated as stochastic processes. The subsequent steps of translation and turnover of protein and mRNA have been left as deterministic ones, since the numbers of molecules in these processes are large.

We suggest that if the model is well-behaved with the critical DNA-binding step as a stochastic process, then the remaining steps can be left as deterministic without compromising the reliability of the model. Three quite different time scales arise in the model. The binding and dissociation of the transcription factors to DNA sites occur on a fast time scale, as discussed below. We introduce an (artificial) parameter *ε *with dimensions of *time *to adjust the time scales for these events and to explore the limit *ε *→ 0. In our Numerical Tests section we vary *ε*; for several numerical simulations we use a value that corresponds to a relatively high rate of binding and dissociation, as explained in the Model section below. Under these conditions the results are essentially indistinguishable from the simulations for a time-averaged deterministic model which is obtained in the limit *ε *→ 0. We subsequently show that the model is well-behaved even for binding rates that are at least 1000-fold slower.

The second significant time scale is given by the periods of the individual ultradian oscillators, which are of the order of a few hours. The critical parameters for these oscillations are those describing the half-lives of mRNA, proteins, and protein complexes. Following our numerical tests, we conduct a brief exploratory analysis of the range of periods of our "primary" oscillators.

The third time scale is, of course, the circadian rhythm time scale, which in our model arises from an interaction of two of the simpler ultradian oscillators of slightly different frequencies. Natural selection could explain why pairs of frequencies leading to the right "beats" have emerged in the course of evolution. In fact, the common occurrence of ultradian oscillators would make it easy for evolution to produce circadian rhythms out of different components in different organisms, as is actually observed [4]. This mechanism has the added advantages of robustness and easy adaptability (the period of the beat will change with minor adjustments of the frequency ratio between the two primary oscillators, but this ratio could stay quite stable even if the parameters involved varied with external conditions such as temperature). A power spectrum analysis presented below demonstrates the robustness of the model with respect to the parameter *ε*. We mention that power spectra could be used to analyze observational data for a potential validation of the model. First steps in this direction were taken in [12].

## The model

Our model involves TTOs contained in a single cell. As described in [4], the model comprises two ultradian "primary" oscillators whose protein products are coupled to drive a circadian rhythm. For simplicity, the two coupled primary oscillators are essentially identical, with only their frequencies different, since the critical feature is the ability to couple TTOs through known molecular processes (formation of transcriptional-regulatory protein heterodimers). Therefore, the key question regarding the ability of a stochastic process to describe stable circadian oscillators can be addressed in terms of one primary oscillator. In this system, two genes (DNA sites) are transcribed into mRNA, and this process is the origin of the following chemical dynamics.

• Transcription by gene 1 occurs when site 1 (its regulatory region) is unoccupied. Its state is given by a random variable *X*_1_, so that

*X*_1 _= 0 if site 1 is empty; *X*_1 _= 1 if site 1 is occupied by *D*_2 _(see below)

• When gene 1 is active it produces mRNA (measured in molecules per cell, *R*_1_) at a constant rate *k*_13_. These molecules undergo first-order decay with a rate constant *k*_14_.

• The mRNA molecules are translated into protein *P*_1_, which: (a) decays at rate constant *k*_16_, (b) forms homodimers *D*_1 _at rate *k*_17_, and (c) forms heterodimers *D*_13 _with proteins *P*_3 _from a third gene (see below) with a rate constant *k*_61_.

• The homodimer *D*_1 _binds to site 2, and thereby activates the transciption of gene 2. The state of gene 2 is given by the value of a random variable *Y*_1 _so that

*Y*_1 _= 0 if site 2 is empty, and *Y*_1 _= 1 if site 2 is occupied by *D*_1_.

• Transcription of gene 2 and translation of its mRNA into protein *P*_2_, which forms homodimer *D*_2_, which in turn feeds back to inhibit gene 1 (above). In addition, the *P*_2 _molecules decay with a certain (biological) half-life.

• These linked reactions generate a TTO for an appropriate choice of parameters. The parameters used in our subsequent calculations are listed in Table 1. Our model entails gene 1 being inhibited by homodimer *D*_2 _and gene 2 being activated by homodimer *D*_1_. This is the mechanism leading to *primary *oscillations.

**Table 1:** Parameters. The dimensions are [*k*_13_] = *hr*^-1^, [*k*_14_] = (*nr*. × *hr*)^-1^, [*k*_17_] = (*nr*.^2 ^× *hr*)^-1^, [*r*] = 1 etc. We assign [*ε*] = *hr*, so that *r, s*,... become dimensionless

Parameter	Value
*k*_13_	1800
*k*_14_	3.2
*k*_15_	700
*k*_16_	4
*k*_17_	3.6 × 10^-4^
*k*_18_	15
*k*_25_	1400
*k*_27_	10^-4^
*k*_28_	5
*k*_53_	500
*k*_54_	0.8
*k*_57_	6.8 × 10^-4^
*k*_58_	3
*k*_61_	5 × 10^-6^
*k*_62_	0.3
*r*	25
*s*	5000
*q*	5500
*δ*	1.125

We denote by *R*_*i*_, *P*_*i*_, *D*_*i*_, *i *= 1, 2 the concentrations of the mRNA, the translated protein and the homodimer produced by site *i*. The above scenario is then summarized in the following system of stochastic differential equations (only two of the equations contain the random variables *X*_1 _and *Y*_1 _explicitly, but all dependent variable are then random variables of necessity). The parameters *k*_13 _etc. have the same meaning as in Ref. [4], and we have kept the notation used there; this explains the unconventional numbering (some of the equations from the reference, and hence some of the parameters, are no longer needed).

R′1=k13(1−X1)−k14R1     (1)
 MathType@MTEF@5@5@+=feaafiart1ev1aaatCvAUfKttLearuWrP9MDH5MBPbIqV92AaeXatLxBI9gBaebbnrfifHhDYfgasaacH8akY=wiFfYdH8Gipec8Eeeu0xXdbba9frFj0=OqFfea0dXdd9vqai=hGuQ8kuc9pgc9s8qqaq=dirpe0xb9q8qiLsFr0=vr0=vr0dc8meaabaqaciaacaGaaeqabaqabeGadaaakeaacuWGsbGugaqbamaaBaaaleaacqaIXaqmaeqaaOGaeyypa0Jaem4AaS2aaSbaaSqaaiabigdaXiabiodaZaqabaGccqGGOaakcqaIXaqmcqGHsislcqWGybawdaWgaaWcbaGaeGymaedabeaakiabcMcaPiabgkHiTiabdUgaRnaaBaaaleaacqaIXaqmcqaI0aanaeqaaOGaemOuai1aaSbaaSqaaiabigdaXaqabaGccaWLjaGaaCzcamaabmaabaGaeGymaedacaGLOaGaayzkaaaaaa@43F0@

P′1=k15R1−k16P1−2k17P12+2k18D1−k61P1P3+k62D13     (2)
 MathType@MTEF@5@5@+=feaafiart1ev1aaatCvAUfKttLearuWrP9MDH5MBPbIqV92AaeXatLxBI9gBaebbnrfifHhDYfgasaacH8akY=wiFfYdH8Gipec8Eeeu0xXdbba9frFj0=OqFfea0dXdd9vqai=hGuQ8kuc9pgc9s8qqaq=dirpe0xb9q8qiLsFr0=vr0=vr0dc8meaabaqaciaacaGaaeqabaqabeGadaaakeaacuWGqbaugaqbamaaBaaaleaacqaIXaqmaeqaaOGaeyypa0Jaem4AaS2aaSbaaSqaaiabigdaXiabiwda1aqabaGccqWGsbGudaWgaaWcbaGaeGymaedabeaakiabgkHiTiabdUgaRnaaBaaaleaacqaIXaqmcqaI2aGnaeqaaOGaemiuaa1aaSbaaSqaaiabigdaXaqabaGccqGHsislcqaIYaGmcqWGRbWAdaWgaaWcbaGaeGymaeJaeG4naCdabeaakiabdcfaqnaaDaaaleaacqaIXaqmaeaacqaIYaGmaaGccqGHRaWkcqaIYaGmcqWGRbWAdaWgaaWcbaGaeGymaeJaeGioaGdabeaakiabdseaenaaBaaaleaacqaIXaqmaeqaaOGaeyOeI0Iaem4AaS2aaSbaaSqaaiabiAda2iabigdaXaqabaGccqWGqbaudaWgaaWcbaGaeGymaedabeaakiabdcfaqnaaBaaaleaacqaIZaWmaeqaaOGaey4kaSIaem4AaS2aaSbaaSqaaiabiAda2iabikdaYaqabaGccqWGebardaWgaaWcbaGaeGymaeJaeG4mamdabeaakiaaxMaacaWLjaWaaeWaaeaacqaIYaGmaiaawIcacaGLPaaaaaa@6123@

D′1=k17P12−k18D1     (3)
 MathType@MTEF@5@5@+=feaafiart1ev1aaatCvAUfKttLearuWrP9MDH5MBPbIqV92AaeXatLxBI9gBaebbnrfifHhDYfgasaacH8akY=wiFfYdH8Gipec8Eeeu0xXdbba9frFj0=OqFfea0dXdd9vqai=hGuQ8kuc9pgc9s8qqaq=dirpe0xb9q8qiLsFr0=vr0=vr0dc8meaabaqaciaacaGaaeqabaqabeGadaaakeaacuWGebargaqbamaaBaaaleaacqaIXaqmaeqaaOGaeyypa0Jaem4AaS2aaSbaaSqaaiabigdaXiabiEda3aqabaGccqWGqbaudaqhaaWcbaGaeGymaedabaGaeGOmaidaaOGaeyOeI0Iaem4AaS2aaSbaaSqaaiabigdaXiabiIda4aqabaGccqWGebardaWgaaWcbaGaeGymaedabeaakiaaxMaacaWLjaWaaeWaaeaacqaIZaWmaiaawIcacaGLPaaaaaa@4120@

R′2=k13Y1−k14R2     (4)
 MathType@MTEF@5@5@+=feaafiart1ev1aaatCvAUfKttLearuWrP9MDH5MBPbIqV92AaeXatLxBI9gBaebbnrfifHhDYfgasaacH8akY=wiFfYdH8Gipec8Eeeu0xXdbba9frFj0=OqFfea0dXdd9vqai=hGuQ8kuc9pgc9s8qqaq=dirpe0xb9q8qiLsFr0=vr0=vr0dc8meaabaqaciaacaGaaeqabaqabeGadaaakeaacuWGsbGugaqbamaaBaaaleaacqaIYaGmaeqaaOGaeyypa0Jaem4AaS2aaSbaaSqaaiabigdaXiabiodaZaqabaGccqWGzbqwdaWgaaWcbaGaeGymaedabeaakiabgkHiTiabdUgaRnaaBaaaleaacqaIXaqmcqaI0aanaeqaaOGaemOuai1aaSbaaSqaaiabikdaYaqabaGccaWLjaGaaCzcamaabmaabaGaeGinaqdacaGLOaGaayzkaaaaaa@406D@

P′2=k25R2−k16P2−2k27P22+2k28D2     (5)
 MathType@MTEF@5@5@+=feaafiart1ev1aaatCvAUfKttLearuWrP9MDH5MBPbIqV92AaeXatLxBI9gBaebbnrfifHhDYfgasaacH8akY=wiFfYdH8Gipec8Eeeu0xXdbba9frFj0=OqFfea0dXdd9vqai=hGuQ8kuc9pgc9s8qqaq=dirpe0xb9q8qiLsFr0=vr0=vr0dc8meaabaqaciaacaGaaeqabaqabeGadaaakeaacuWGqbaugaqbamaaBaaaleaacqaIYaGmaeqaaOGaeyypa0Jaem4AaS2aaSbaaSqaaiabikdaYiabiwda1aqabaGccqWGsbGudaWgaaWcbaGaeGOmaidabeaakiabgkHiTiabdUgaRnaaBaaaleaacqaIXaqmcqaI2aGnaeqaaOGaemiuaa1aaSbaaSqaaiabikdaYaqabaGccqGHsislcqaIYaGmcqWGRbWAdaWgaaWcbaGaeGOmaiJaeG4naCdabeaakiabdcfaqnaaDaaaleaacqaIYaGmaeaacqaIYaGmaaGccqGHRaWkcqaIYaGmcqWGRbWAdaWgaaWcbaGaeGOmaiJaeGioaGdabeaakiabdseaenaaBaaaleaacqaIYaGmaeqaaOGaaCzcaiaaxMaadaqadaqaaiabiwda1aGaayjkaiaawMcaaaaa@509D@

D′2=k27P22−k28D2     (6)
 MathType@MTEF@5@5@+=feaafiart1ev1aaatCvAUfKttLearuWrP9MDH5MBPbIqV92AaeXatLxBI9gBaebbnrfifHhDYfgasaacH8akY=wiFfYdH8Gipec8Eeeu0xXdbba9frFj0=OqFfea0dXdd9vqai=hGuQ8kuc9pgc9s8qqaq=dirpe0xb9q8qiLsFr0=vr0=vr0dc8meaabaqaciaacaGaaeqabaqabeGadaaakeaacuWGebargaqbamaaBaaaleaacqaIYaGmaeqaaOGaeyypa0Jaem4AaS2aaSbaaSqaaiabikdaYiabiEda3aqabaGccqWGqbaudaqhaaWcbaGaeGOmaidabaGaeGOmaidaaOGaeyOeI0Iaem4AaS2aaSbaaSqaaiabikdaYiabiIda4aqabaGccqWGebardaWgaaWcbaGaeGOmaidabeaakiaaxMaacaWLjaWaaeWaaeaacqaI2aGnaiaawIcacaGLPaaaaaa@4130@

The last two terms in the second equation reflect the combination of proteins *P*_1 _and *P*_3 _(which is produced by the second primary oscillator) to form the heterodimer *D*_13_. This heterodimer in turn breaks down into pairs *P*_1 _and *P*_3 _at rate constant *k*_62_.

The second primary oscillator is given by a nearly identical set of equations, except that the periods of the oscillations are slightly different. This can, of course, be achieved by changing the parameters in many ways, but the simplest method is to have the two TTOs identical in nature but with different time scales. To do this we simply multiply each right hand side by a fixed constant *δ *> 0, where *δ *is close (but not identical) to one. For example, the first equation of the second oscillator will read

R′3
 MathType@MTEF@5@5@+=feaafiart1ev1aaatCvAUfKttLearuWrP9MDH5MBPbIqV92AaeXatLxBI9gBaebbnrfifHhDYfgasaacH8akY=wiFfYdH8Gipec8Eeeu0xXdbba9frFj0=OqFfea0dXdd9vqai=hGuQ8kuc9pgc9s8qqaq=dirpe0xb9q8qiLsFr0=vr0=vr0dc8meaabaqaciaacaGaaeqabaqabeGadaaakeaacuWGsbGugaqbamaaBaaaleaacqaIZaWmaeqaaaaa@2F05@ = *δ*(*k*_13_(1 - *X*_2_) - *k*_14_*R*_3_).

The parameters chosen reflect, where available, reasonable choices of known molecular processes. The critical ones for establishing the periods of the primary oscillators are the decay times of the mRNAs and proteins. For the former, a half-life of 13–17 minutes and for the latter, 4–17 minutes generate ultradian oscillations in the model. The values used in the simulation are given in Table 1.

The coupling between the two sites communicating in each oscillator is, of course, provided by the random variables *X*_*i*_, *Y*_*i*_. The times for which these random variables stay constant are assumed to be exponentially distributed. For example,

*Prob*{*X*_1 _= 0 in (*t, t + h*)|*X*_1_(*t*) = 0} = exp (-*D*_2_(*t*)*h*/*ε*) + *o*(*h*),

*Prob*{*X*_1 _= 1 in (*t, t + h*)|*X*_1_(*t*) = 1} = exp (-*rh*/*ε*) + *o*(*h*)

while

*Prob*{*Y*_1 _= 0 in (*t, t + h*)|*Y*_1_(*t*) = 0} = exp (-*D*_1_(*t*)*h*/*ε*) + *o*(*h*),

*Prob*{*Y*_1 _= 1 in (*t, t + h*)|*Y*_1_(*t*) = 1} = exp (-*st*/*ε*) + *o*(*h*).

Here *ε *is a time scaling parameter, introduced for convenience to exploit the fact that the binding and unbinding of the homodimers occurs on a faster time scale than the remaining processes. The constants *r *and *s *measure, relative to the scale *ε*, the average times for which the sites will remain occupied. As this is an internal parameter of the site it should not depend on the states of the rest of the system (like, for example, the dimer concentrations).

**Figure 1:**
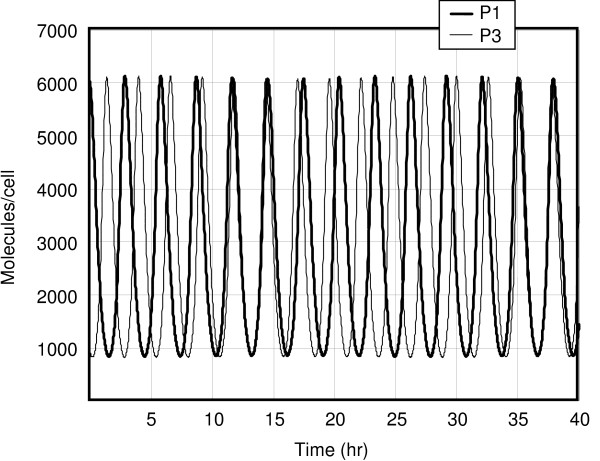
The time evolution of the proteins *P*_1 _and *P*_3 _according to the time-averaged model.

**Figure 2:**
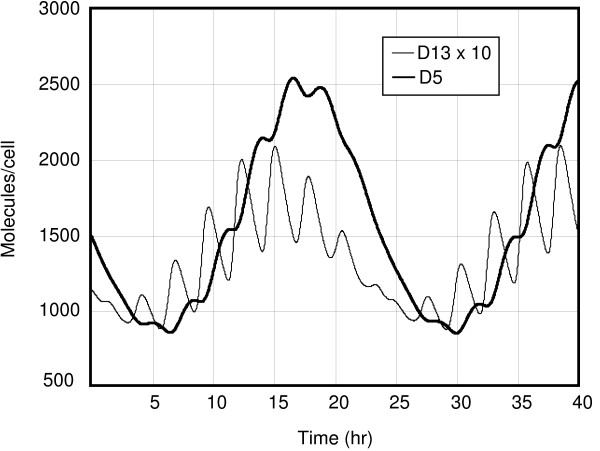
The time evolution of the heterodimer *D*_13 _and the homodimer *D*_5 _according to the time-averaged model.

**Figure 3:**
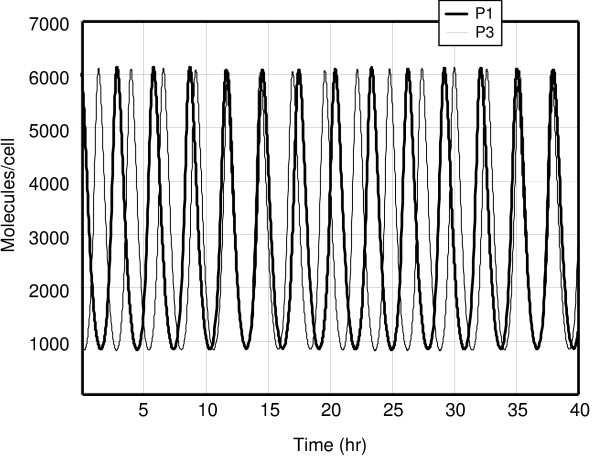
The time evolution of the proteins *P*_1 _and *P*_3 _according to the stochastic model.

**Figure 4:**
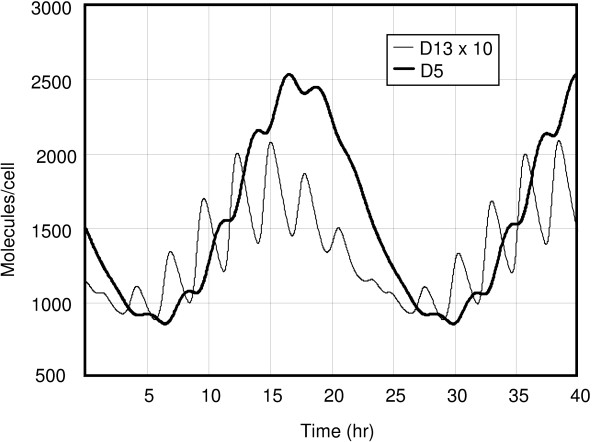
The time evolution of the heterodimer *D*_13 _and the homodimer *D*_5 _according to the stochastic model.

**Figure 5:**
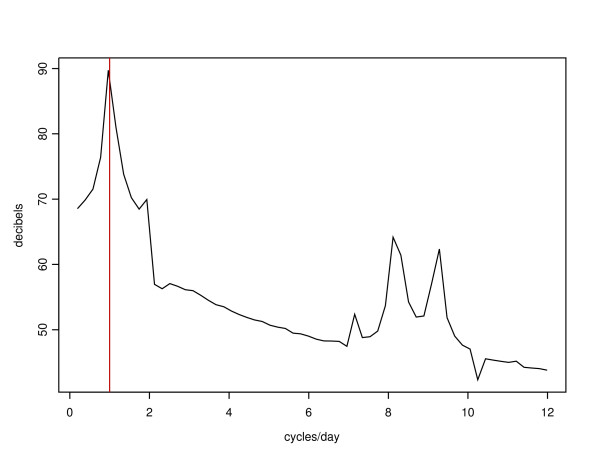
Power spectra for *D*_5 _when *ε *= 2.8 × 10^-5 ^(no smoothing).

**Figure 6:**
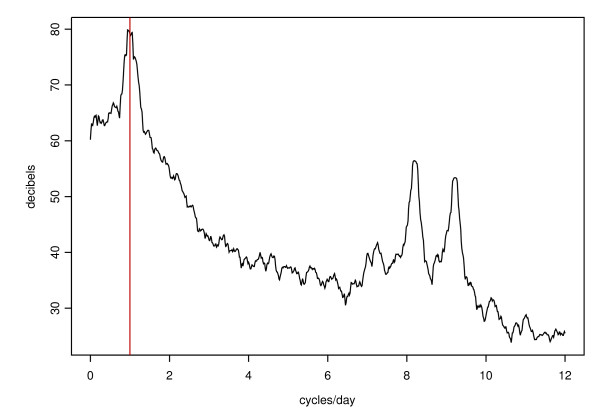
Power spectra for *D*_5 _when *ε *= 2.8 × 10^-2 ^(smoothed with a Daniell filter of length 11).

We use *ε *to gauge the rate constant for binding of the transcriptional-regulatory proteins (D1, D2) to the binding sites on the relevant genes. Experimental work has shown that the second-order rate constant for the binding of transcription-regulating proteins to DNA can be 100 to 1000 times greater than the maximum rate predicted for three-dimensional diffusion [13,14]. With transcription-regulating protein concentrations measured in molecules/nucleus, using the experimental rate constant for binding of the lac repressor to its cognate DNA [10^-10^(Msec)^-1^], and assuming that a small eukaryotic nucleus has an effective volume of 40% of its total volume, this suggests a value for *ε *of 0.10 seconds (2.8 × 10^-5 ^hours). This can be interpreted as the time required for a binding event when Dl or D2 is present at 1 molecule/nucleus. At higher concentrations (of D1 or D2), this time will shorten proportionately. The average "free" time of the binding site for *D*_2 _is thus *ε*/*D*_2_, and the average "occupied" time is *ε*/*r*. Their quotient is independent of *ε*, but will change with the homodimer concentration *D*_2_. Similar interpretations apply for *X*_2 _and *Y*_2 _and the random variables associated with the second primary oscillator. We have used the value *ε *= 0.1 sec for producing most of the numerical simulations in our Numerical Tests Section below (Figures 1, 2, 3, 4). However, as shown in Figures 5 and 6, an *ε *of 1000 times greater value (corresponding to a 1000-fold slower rate of binding) yields effectively the same power spectrum for the circadian model. This is comparable to the observation by Forger and Peskin that in their model for mammalian circadian rhythms the on/off times need to be in the order of seconds.

The average times for which a dimer stays bound (*ε*/*r*, *ε*/*s*, etc.) are independent of the state of the system. In contrast, the "free" times are inversely proportional to the concentration of the attaching homodimer. In one of our simulations we use *r *= 25 and *ε *= 10^-1^sec (which corresponds to εr=1250
 MathType@MTEF@5@5@+=feaafiart1ev1aaatCvAUfKttLearuWrP9MDH5MBPbIqV92AaeXatLxBI9gBaebbnrfifHhDYfgasaacH8akY=wiFfYdH8Gipec8Eeeu0xXdbba9frFj0=OqFfea0dXdd9vqai=hGuQ8kuc9pgc9s8qqaq=dirpe0xb9q8qiLsFr0=vr0=vr0dc8meaabaqaciaacaGaaeqabaqabeGadaaakeaadaWcaaqaaGGaciab=v7aLbqaaiabdkhaYbaacqGH9aqpdaWcaaqaaiabigdaXaqaaiabikdaYiabiwda1iabicdaWaaaaaa@34B5@ sec, or an average of 900,000 binding events per hour). We shall see that the corresponding stochastic simulation compares well with a limiting scenario for which *ε *= 0. Before we describe this limiting scenario in detail we present the remaining equations making up the complete oscillatory system.

As stated earlier, the protein products *P*_1 _and *P*_3 _of the first and second primary oscillators combine to produce the heterodimer *D*_13_. As formulated in the model, this heterodimer binds to the regulatory site of a fifth gene and activates it for transcription (other constructs, involving other heterodimeric products of the two primary oscillators, and either stimulation or inhibition of transcription of the fifth gene, could also be used). Transcription, translation, and dimerization of the protein product of gene 5 yields the product *D*_5_, which is the primary circadian output of the model (although all variables show circadian behaviour to a greater or less extent, as seen in the graphical results).

The corresponding system is

D′13=k61P1P3−k62D13     (7)
 MathType@MTEF@5@5@+=feaafiart1ev1aaatCvAUfKttLearuWrP9MDH5MBPbIqV92AaeXatLxBI9gBaebbnrfifHhDYfgasaacH8akY=wiFfYdH8Gipec8Eeeu0xXdbba9frFj0=OqFfea0dXdd9vqai=hGuQ8kuc9pgc9s8qqaq=dirpe0xb9q8qiLsFr0=vr0=vr0dc8meaabaqaciaacaGaaeqabaqabeGadaaakeaacuWGebargaqbamaaBaaaleaacqaIXaqmcqaIZaWmaeqaaOGaeyypa0Jaem4AaS2aaSbaaSqaaiabiAda2iabigdaXaqabaGccqWGqbaudaWgaaWcbaGaeGymaedabeaakiabdcfaqnaaBaaaleaacqaIZaWmaeqaaOGaeyOeI0Iaem4AaS2aaSbaaSqaaiabiAda2iabikdaYaqabaGccqWGebardaWgaaWcbaGaeGymaeJaeG4mamdabeaakiaaxMaacaWLjaWaaeWaaeaacqaI3aWnaiaawIcacaGLPaaaaaa@446C@

R′5=k53X3−k54R5     (8)
 MathType@MTEF@5@5@+=feaafiart1ev1aaatCvAUfKttLearuWrP9MDH5MBPbIqV92AaeXatLxBI9gBaebbnrfifHhDYfgasaacH8akY=wiFfYdH8Gipec8Eeeu0xXdbba9frFj0=OqFfea0dXdd9vqai=hGuQ8kuc9pgc9s8qqaq=dirpe0xb9q8qiLsFr0=vr0=vr0dc8meaabaqaciaacaGaaeqabaqabeGadaaakeaacuWGsbGugaqbamaaBaaaleaacqaI1aqnaeqaaOGaeyypa0Jaem4AaS2aaSbaaSqaaiabiwda1iabiodaZaqabaGccqWGybawdaWgaaWcbaGaeG4mamdabeaakiabgkHiTiabdUgaRnaaBaaaleaacqaI1aqncqaI0aanaeqaaOGaemOuai1aaSbaaSqaaiabiwda1aqabaGccaWLjaGaaCzcamaabmaabaGaeGioaGdacaGLOaGaayzkaaaaaa@4093@

P′5=k15R5−k16P5−2k57P52+2k58D5     (9)
 MathType@MTEF@5@5@+=feaafiart1ev1aaatCvAUfKttLearuWrP9MDH5MBPbIqV92AaeXatLxBI9gBaebbnrfifHhDYfgasaacH8akY=wiFfYdH8Gipec8Eeeu0xXdbba9frFj0=OqFfea0dXdd9vqai=hGuQ8kuc9pgc9s8qqaq=dirpe0xb9q8qiLsFr0=vr0=vr0dc8meaabaqaciaacaGaaeqabaqabeGadaaakeaacuWGqbaugaqbamaaBaaaleaacqaI1aqnaeqaaOGaeyypa0Jaem4AaS2aaSbaaSqaaiabigdaXiabiwda1aqabaGccqWGsbGudaWgaaWcbaGaeGynaudabeaakiabgkHiTiabdUgaRnaaBaaaleaacqaIXaqmcqaI2aGnaeqaaOGaemiuaa1aaSbaaSqaaiabiwda1aqabaGccqGHsislcqaIYaGmcqWGRbWAdaWgaaWcbaGaeGynauJaeG4naCdabeaakiabdcfaqnaaDaaaleaacqaI1aqnaeaacqaIYaGmaaGccqGHRaWkcqaIYaGmcqWGRbWAdaWgaaWcbaGaeGynauJaeGioaGdabeaakiabdseaenaaBaaaleaacqaI1aqnaeqaaOGaaCzcaiaaxMaadaqadaqaaiabiMda5aGaayjkaiaawMcaaaaa@50CD@

D′5=k57P52−k58D5,     (10)
 MathType@MTEF@5@5@+=feaafiart1ev1aaatCvAUfKttLearuWrP9MDH5MBPbIqV92AaeXatLxBI9gBaebbnrfifHhDYfgasaacH8akY=wiFfYdH8Gipec8Eeeu0xXdbba9frFj0=OqFfea0dXdd9vqai=hGuQ8kuc9pgc9s8qqaq=dirpe0xb9q8qiLsFr0=vr0=vr0dc8meaabaqaciaacaGaaeqabaqabeGadaaakeaacuWGebargaqbamaaBaaaleaacqaI1aqnaeqaaOGaeyypa0Jaem4AaS2aaSbaaSqaaiabiwda1iabiEda3aqabaGccqWGqbaudaqhaaWcbaGaeGynaudabaGaeGOmaidaaOGaeyOeI0Iaem4AaS2aaSbaaSqaaiabiwda1iabiIda4aqabaGccqWGebardaWgaaWcbaGaeGynaudabeaakiabcYcaSiaaxMaacaWLjaWaaeWaaeaacqaIXaqmcqaIWaamaiaawIcacaGLPaaaaaa@4312@

and

Prob{X3=0 in (t,t+h)|X3(t)=0}=exp⁡(−D13(t)εh)+o(h),Prob{X3=1 in (t,t+h)|X3(t)=1}=exp⁡(−qεh)+o(h).
 MathType@MTEF@5@5@+=feaafiart1ev1aaatCvAUfKttLearuWrP9MDH5MBPbIqV92AaeXatLxBI9gBaebbnrfifHhDYfgasaacH8akY=wiFfYdH8Gipec8Eeeu0xXdbba9frFj0=OqFfea0dXdd9vqai=hGuQ8kuc9pgc9s8qqaq=dirpe0xb9q8qiLsFr0=vr0=vr0dc8meaabaqaciaacaGaaeqabaqabeGadaaakeaafaqabeGabaaabaacbiGae8huaaLae8NCaiNaem4Ba8MaemOyaiMaei4EaSNaemiwaG1aaSbaaSqaaiabiodaZaqabaGccqGH9aqpcqaIWaamcqqGGaaicqqGPbqAcqqGUbGBcqqGGaaicqGGOaakcqWG0baDcqGGSaalcqWG0baDcqGHRaWkcqWGObaAcqGGPaqkcqGG8baFcqWGybawdaWgaaWcbaGaeG4mamdabeaakiabcIcaOiabdsha0jabcMcaPiabg2da9iabicdaWiabc2ha9jabg2da9iGbcwgaLjabcIha4jabcchaWnaabmaabaWaaSaaaeaacqGHsislcqWGebardaWgaaWcbaGaeGymaeJaeG4mamdabeaakiabcIcaOiabdsha0jabcMcaPaqaaGGaciab+v7aLbaacqWGObaAaiaawIcacaGLPaaacqGHRaWkcqWGVbWBcqGGOaakcqWGObaAcqGGPaqkcqGGSaalaeaacqWFqbaucqWFYbGCcqWGVbWBcqWGIbGycqGG7bWEcqWGybawdaWgaaWcbaGaeG4mamdabeaakiabg2da9iabigdaXiabbccaGiabbMgaPjabb6gaUjabbccaGiabcIcaOiabdsha0jabcYcaSiabdsha0jabgUcaRiabdIgaOjabcMcaPiabcYha8jabdIfaynaaBaaaleaacqaIZaWmaeqaaOGaeiikaGIaemiDaqNaeiykaKIaeyypa0JaeGymaeJaeiyFa0Naeyypa0JagiyzauMaeiiEaGNaeiiCaa3aaeWaaeaadaWcaaqaaiabgkHiTiabdghaXbqaaiab+v7aLbaacqWGObaAaiaawIcacaGLPaaacqGHRaWkcqWGVbWBcqGGOaakcqWGObaAcqGGPaqkcqGGUaGlaaaaaa@9985@

## The time-averaged deterministic model

We employ renewal reward theory (see [15]) to derive a system of ordinary differential equations which replaces (1–6) by a "time-averaged" system in the limit *ε *→ 0. To this end, note first that if *D*_2 _were independent of time, the time average of *X*_1_(*t*) over "macroscopic" time intervals (i.e., intervals of scale much larger than *ε*) is D2r+D2
 MathType@MTEF@5@5@+=feaafiart1ev1aaatCvAUfKttLearuWrP9MDH5MBPbIqV92AaeXatLxBI9gBaebbnrfifHhDYfgasaacH8akY=wiFfYdH8Gipec8Eeeu0xXdbba9frFj0=OqFfea0dXdd9vqai=hGuQ8kuc9pgc9s8qqaq=dirpe0xb9q8qiLsFr0=vr0=vr0dc8meaabaqaciaacaGaaeqabaqabeGadaaakeaadaWcaaqaaiabdseaenaaBaaaleaacqaIYaGmaeqaaaGcbaGaemOCaiNaey4kaSIaemiraq0aaSbaaSqaaiabikdaYaqabaaaaaaa@3373@. The corresponding average of 1 - *X*_1_(*t*) is then rr+D2
 MathType@MTEF@5@5@+=feaafiart1ev1aaatCvAUfKttLearuWrP9MDH5MBPbIqV92AaeXatLxBI9gBaebbnrfifHhDYfgasaacH8akY=wiFfYdH8Gipec8Eeeu0xXdbba9frFj0=OqFfea0dXdd9vqai=hGuQ8kuc9pgc9s8qqaq=dirpe0xb9q8qiLsFr0=vr0=vr0dc8meaabaqaciaacaGaaeqabaqabeGadaaakeaadaWcaaqaaiabdkhaYbqaaiabdkhaYjabgUcaRiabdseaenaaBaaaleaacqaIYaGmaeqaaaaaaaa@32A7@. Renewal reward theory implies that this intuition is mathematically accurate.

Specifically, define a cycle to consist of a period of unoccupied time followed by a period of occupied time. The cycle ends with detachment. The period of unoccupied time is exponentially distributed with mean *ε*/*D*_2_. Suppose, in the language of renewal reward theory, that no reward is received during this time. The following occupied part of the cycle is exponentially distributed with mean *ε*/*r*, and we assume that the reward associated with this period is exactly equal to the amount of occupied time. Then, by renewal reward theory, the long-term average reward (i.e., the proportion of occupied time) is with probability 1 equal to *E*(*R*)/*E*(*L*) where *E*(*R*) is the expected reward during a cycle and *E*(*L*) is the expected length of a cycle. In the case under consideration

*E*(*R*) = *ε*/*r*, *E*(*L*) = *ε*/*r *+ *ε*/*D*_2_,

so the long-term time average of *X*_1_(*t*) is *D*_2_/(*r *+ *D*_2_), i.e., lim_*ε*→0 _*X*_1*ε*_(*t*) = D2r+D2
 MathType@MTEF@5@5@+=feaafiart1ev1aaatCvAUfKttLearuWrP9MDH5MBPbIqV92AaeXatLxBI9gBaebbnrfifHhDYfgasaacH8akY=wiFfYdH8Gipec8Eeeu0xXdbba9frFj0=OqFfea0dXdd9vqai=hGuQ8kuc9pgc9s8qqaq=dirpe0xb9q8qiLsFr0=vr0=vr0dc8meaabaqaciaacaGaaeqabaqabeGadaaakeaadaWcaaqaaiabdseaenaaBaaaleaacqaIYaGmaeqaaaGcbaGaemOCaiNaey4kaSIaemiraq0aaSbaaSqaaiabikdaYaqabaaaaaaa@3373@ (here, we denote the random variables *X*_*i *_as *X*_*iε *_to emphasize the dependence on *ε*). This time average will hold over any time interval over which *D*_2 _is constant or changes sufficiently slowly. In this time-averaged system Eqns. (1,4) then become

R′1=k13rr+D2−k14R1     (11)
 MathType@MTEF@5@5@+=feaafiart1ev1aaatCvAUfKttLearuWrP9MDH5MBPbIqV92AaeXatLxBI9gBaebbnrfifHhDYfgasaacH8akY=wiFfYdH8Gipec8Eeeu0xXdbba9frFj0=OqFfea0dXdd9vqai=hGuQ8kuc9pgc9s8qqaq=dirpe0xb9q8qiLsFr0=vr0=vr0dc8meaabaqaciaacaGaaeqabaqabeGadaaakeaacuWGsbGugaqbamaaBaaaleaacqaIXaqmaeqaaOGaeyypa0Jaem4AaS2aaSbaaSqaaiabigdaXiabiodaZaqabaGcdaWcaaqaaiabdkhaYbqaaiabdkhaYjabgUcaRiabdseaenaaBaaaleaacqaIYaGmaeqaaaaakiabgkHiTiabdUgaRnaaBaaaleaacqaIXaqmcqaI0aanaeqaaOGaemOuai1aaSbaaSqaaiabigdaXaqabaGccaWLjaGaaCzcamaabmaabaGaeGymaeJaeGymaedacaGLOaGaayzkaaaaaa@44F7@

R′2=k13D1s+D1−k14R2     (12)
 MathType@MTEF@5@5@+=feaafiart1ev1aaatCvAUfKttLearuWrP9MDH5MBPbIqV92AaeXatLxBI9gBaebbnrfifHhDYfgasaacH8akY=wiFfYdH8Gipec8Eeeu0xXdbba9frFj0=OqFfea0dXdd9vqai=hGuQ8kuc9pgc9s8qqaq=dirpe0xb9q8qiLsFr0=vr0=vr0dc8meaabaqaciaacaGaaeqabaqabeGadaaakeaacuWGsbGugaqbamaaBaaaleaacqaIYaGmaeqaaOGaeyypa0Jaem4AaS2aaSbaaSqaaiabigdaXiabiodaZaqabaGcdaWcaaqaaiabdseaenaaBaaaleaacqaIXaqmaeqaaaGcbaGaem4CamNaey4kaSIaemiraq0aaSbaaSqaaiabigdaXaqabaaaaOGaeyOeI0Iaem4AaS2aaSbaaSqaaiabigdaXiabisda0aqabaGccqWGsbGudaWgaaWcbaGaeGOmaidabeaakiaaxMaacaWLjaWaaeWaaeaacqaIXaqmcqaIYaGmaiaawIcacaGLPaaaaaa@45C7@

and the remaining equations stay the same. Similarly, Equation (8) becomes

R′5=k53D13(q+D13)−k54R5.
 MathType@MTEF@5@5@+=feaafiart1ev1aaatCvAUfKttLearuWrP9MDH5MBPbIqV92AaeXatLxBI9gBaebbnrfifHhDYfgasaacH8akY=wiFfYdH8Gipec8Eeeu0xXdbba9frFj0=OqFfea0dXdd9vqai=hGuQ8kuc9pgc9s8qqaq=dirpe0xb9q8qiLsFr0=vr0=vr0dc8meaabaqaciaacaGaaeqabaqabeGadaaakeaacuWGsbGugaqbamaaBaaaleaacqaI1aqnaeqaaOGaeyypa0Jaem4AaS2aaSbaaSqaaiabiwda1iabiodaZaqabaGcdaWcaaqaaiabdseaenaaBaaaleaacqaIXaqmcqaIZaWmaeqaaaGcbaGaeiikaGIaemyCaeNaey4kaSIaemiraq0aaSbaaSqaaiabigdaXiabiodaZaqabaGccqGGPaqkaaGaeyOeI0Iaem4AaS2aaSbaaSqaaiabiwda1iabisda0aqabaGccqWGsbGudaWgaaWcbaGaeGynaudabeaakiabc6caUaaa@45AE@

This intuitive argument is not rigorous. As is transparent from the equations for the primary oscillators, all the dependent variables are random variables with time fluctuations at time scale *ε*. In particular, *D*_1 _and *D*_2 _(and likewise *D*_3 _and *D*_4_) experience stochastic fluctuations in their third derivatives (R′1
 MathType@MTEF@5@5@+=feaafiart1ev1aaatCvAUfKttLearuWrP9MDH5MBPbIqV92AaeXatLxBI9gBaebbnrfifHhDYfgasaacH8akY=wiFfYdH8Gipec8Eeeu0xXdbba9frFj0=OqFfea0dXdd9vqai=hGuQ8kuc9pgc9s8qqaq=dirpe0xb9q8qiLsFr0=vr0=vr0dc8meaabaqaciaacaGaaeqabaqabeGadaaakeaacuWGsbGugaqbamaaBaaaleaacqaIXaqmaeqaaaaa@2F01@ experiences random jumps, as does P″1
 MathType@MTEF@5@5@+=feaafiart1ev1aaatCvAUfKttLearuWrP9MDH5MBPbIqV92AaeXatLxBI9gBaebbnrfifHhDYfgasaacH8akY=wiFfYdH8Gipec8Eeeu0xXdbba9frFj0=OqFfea0dXdd9vqai=hGuQ8kuc9pgc9s8qqaq=dirpe0xb9q8qiLsFr0=vr0=vr0dc8meaabaqaciaacaGaaeqabaqabeGadaaakeaacuWGqbaugaGbamaaBaaaleaacqaIXaqmaeqaaaaa@2EFE@, and as does D‴1
 MathType@MTEF@5@5@+=feaafiart1ev1aaatCvAUfKttLearuWrP9MDH5MBPbIqV92AaeXatLxBI9gBaebbnrfifHhDYfgasaacH8akY=wiFfYdH8Gipec8Eeeu0xXdbba9frFj0=OqFfea0dXdd9vqai=hGuQ8kuc9pgc9s8qqaq=dirpe0xb9q8qiLsFr0=vr0=vr0dc8meaabaqaciaacaGaaeqabaqabeGadaaakeaacuWGebargaGeamaaBaaaleaacqaIXaqmaeqaaaaa@2EF2@). The integration process involved in the computation of *D*_*i*_, (*i *= 1, 2) will average out these fluctuations, so that *D*_*i *_will indeed vary more slowly than, say, *R*_*i*_. An argument based on the Arzelà-Ascoli Theorem can be used to translate these observations into a mathematical proof.

To this end we denote by *R*_1*ε*_, *P*_1*ε*_, *D*_1*ε *_etc. the solution of (1–6) for some *ε *> 0 and given initial values *R*_1_(0), *P*_1_(0),..., and denote by *R*_1_, *P*_1_, *D*_1 _etc. the solution of Eqns. (11, 12) ff. for the same initial values.

We prove

**Proposition 1 ***Almost surely for all t *> 0,

lim⁡ε→0R1ε(t)=R1(t)lim⁡ε→0P1ε(t)=P1(t)lim⁡ε→0D1ε(t)=D1(t)lim⁡ε→0R2ε(t)=R2(t)
 MathType@MTEF@5@5@+=feaafiart1ev1aaatCvAUfKttLearuWrP9MDH5MBPbIqV92AaeXatLxBI9gBaebbnrfifHhDYfgasaacH8akY=wiFfYdH8Gipec8Eeeu0xXdbba9frFj0=OqFfea0dXdd9vqai=hGuQ8kuc9pgc9s8qqaq=dirpe0xb9q8qiLsFr0=vr0=vr0dc8meaabaqaciaacaGaaeqabaqabeGadaaakqaabeqaamaaxababaGagiiBaWMaeiyAaKMaeiyBa0galeaaiiGacqWF1oqzcqGHsgIRcqaIWaamaeqaaOGaemOuai1aaSbaaSqaaiabigdaXiab=v7aLbqabaGccqGGOaakcqWG0baDcqGGPaqkcqGH9aqpcqWGsbGudaWgaaWcbaGaeGymaedabeaakiabcIcaOiabdsha0jabcMcaPaqaamaaxababaGagiiBaWMaeiyAaKMaeiyBa0galeaacqWF1oqzcqGHsgIRcqaIWaamaeqaaOGaemiuaa1aaSbaaSqaaiabigdaXiab=v7aLbqabaGccqGGOaakcqWG0baDcqGGPaqkcqGH9aqpcqWGqbaudaWgaaWcbaGaeGymaedabeaakiabcIcaOiabdsha0jabcMcaPaqaamaaxababaGagiiBaWMaeiyAaKMaeiyBa0galeaacqWF1oqzcqGHsgIRcqaIWaamaeqaaOGaemiraq0aaSbaaSqaaiabigdaXiab=v7aLbqabaGccqGGOaakcqWG0baDcqGGPaqkcqGH9aqpcqWGebardaWgaaWcbaGaeGymaedabeaakiabcIcaOiabdsha0jabcMcaPaqaamaaxababaGagiiBaWMaeiyAaKMaeiyBa0galeaacqWF1oqzcqGHsgIRcqaIWaamaeqaaOGaemOuai1aaSbaaSqaaiabikdaYiab=v7aLbqabaGccqGGOaakcqWG0baDcqGGPaqkcqGH9aqpcqWGsbGudaWgaaWcbaGaeGOmaidabeaakiabcIcaOiabdsha0jabcMcaPaaaaa@864D@

*etc*.

*Proof*.

**Step 1**. Consider an arbitrary but fixed time interval [0, *T*] and let (*ε*_*n*_) be a sequence such that *ε*_*n *_→ 0 as *n *→ ∞. For each *n *we consider a realization, again denoted by *R*_1*ε *_etc., of the initial value problem (1–6) ff. with the given fixed initial data.

The resulting functions R1εn
 MathType@MTEF@5@5@+=feaafiart1ev1aaatCvAUfKttLearuWrP9MDH5MBPbIqV92AaeXatLxBI9gBaebbnrfifHhDYfgasaacH8akY=wiFfYdH8Gipec8Eeeu0xXdbba9frFj0=OqFfea0dXdd9vqai=hGuQ8kuc9pgc9s8qqaq=dirpe0xb9q8qiLsFr0=vr0=vr0dc8meaabaqaciaacaGaaeqabaqabeGadaaakeaacqWGsbGudaWgaaWcbaGaeGymaedcciGae8xTdu2aaSbaaWqaaiabd6gaUbqabaaaleqaaaaa@3240@, P1εn
 MathType@MTEF@5@5@+=feaafiart1ev1aaatCvAUfKttLearuWrP9MDH5MBPbIqV92AaeXatLxBI9gBaebbnrfifHhDYfgasaacH8akY=wiFfYdH8Gipec8Eeeu0xXdbba9frFj0=OqFfea0dXdd9vqai=hGuQ8kuc9pgc9s8qqaq=dirpe0xb9q8qiLsFr0=vr0=vr0dc8meaabaqaciaacaGaaeqabaqabeGadaaakeaacqWGqbaudaWgaaWcbaGaeGymaedcciGae8xTdu2aaSbaaWqaaiabd6gaUbqabaaaleqaaaaa@323C@, D1εn
 MathType@MTEF@5@5@+=feaafiart1ev1aaatCvAUfKttLearuWrP9MDH5MBPbIqV92AaeXatLxBI9gBaebbnrfifHhDYfgasaacH8akY=wiFfYdH8Gipec8Eeeu0xXdbba9frFj0=OqFfea0dXdd9vqai=hGuQ8kuc9pgc9s8qqaq=dirpe0xb9q8qiLsFr0=vr0=vr0dc8meaabaqaciaacaGaaeqabaqabeGadaaakeaacqWGebardaWgaaWcbaGaeGymaedcciGae8xTdu2aaSbaaWqaaiabd6gaUbqabaaaleqaaaaa@3224@,... all remain bounded and have (uniformly in *ε*) bounded first derivatives on [0, *T*]. By the Arzelà-Ascoli Theorem, there is a convergent subsequence of *ε*_*n*_, denoted again by *ε*_*n*_. We denote the limits by R˜1
 MathType@MTEF@5@5@+=feaafiart1ev1aaatCvAUfKttLearuWrP9MDH5MBPbIqV92AaeXatLxBI9gBaebbnrfifHhDYfgasaacH8akY=wiFfYdH8Gipec8Eeeu0xXdbba9frFj0=OqFfea0dXdd9vqai=hGuQ8kuc9pgc9s8qqaq=dirpe0xb9q8qiLsFr0=vr0=vr0dc8meaabaqaciaacaGaaeqabaqabeGadaaakeaacuWGsbGugaacamaaBaaaleaacqaIXaqmaeqaaaaa@2F04@, P˜1
 MathType@MTEF@5@5@+=feaafiart1ev1aaatCvAUfKttLearuWrP9MDH5MBPbIqV92AaeXatLxBI9gBaebbnrfifHhDYfgasaacH8akY=wiFfYdH8Gipec8Eeeu0xXdbba9frFj0=OqFfea0dXdd9vqai=hGuQ8kuc9pgc9s8qqaq=dirpe0xb9q8qiLsFr0=vr0=vr0dc8meaabaqaciaacaGaaeqabaqabeGadaaakeaacuWGqbaugaacamaaBaaaleaacqaIXaqmaeqaaaaa@2F00@,.... What we show next is that these limits are solutions of the deterministic limit equations (11,12) ff.

**Step 2**. We write *ε *rather than *ε*_*n *_to simplify the notation. Observe that

R1ε(t)=R1(0)e−k14t+k13∫0t(1−X1ε)(τ)ek14(τ−t)dτ
 MathType@MTEF@5@5@+=feaafiart1ev1aaatCvAUfKttLearuWrP9MDH5MBPbIqV92AaeXatLxBI9gBaebbnrfifHhDYfgasaacH8akY=wiFfYdH8Gipec8Eeeu0xXdbba9frFj0=OqFfea0dXdd9vqai=hGuQ8kuc9pgc9s8qqaq=dirpe0xb9q8qiLsFr0=vr0=vr0dc8meaabaqaciaacaGaaeqabaqabeGadaaakeaacqWGsbGudaWgaaWcbaGaeGymaedcciGae8xTdugabeaakiabcIcaOiabdsha0jabcMcaPiabg2da9iabdkfasnaaBaaaleaacqaIXaqmaeqaaOGaeiikaGIaeGimaaJaeiykaKIaemyzau2aaWbaaSqabeaacqGHsislcqWGRbWAdaWgaaadbaGaeGymaeJaeGinaqdabeaaliabdsha0baakiabgUcaRiabdUgaRnaaBaaaleaacqaIXaqmcqaIZaWmaeqaaOWaa8qmaeaacqGGOaakcqaIXaqmcqGHsislcqWGybawdaWgaaWcbaGaeGymaeJae8xTdugabeaakiabcMcaPiabcIcaOiab=r8a0jabcMcaPiabdwgaLnaaCaaaleqabaGaem4AaS2aaSbaaWqaaiabigdaXiabisda0aqabaWccqGGOaakcqWFepaDcqGHsislcqWG0baDcqGGPaqkaaGccqWGKbazcqWFepaDaSqaaiabicdaWaqaaiabdsha0bqdcqGHRiI8aaaa@62FB@

and

R1(t)=R1(0)e−k14t+k13∫0trr+D2(τ)ek14(τ−t)dτ.
 MathType@MTEF@5@5@+=feaafiart1ev1aaatCvAUfKttLearuWrP9MDH5MBPbIqV92AaeXatLxBI9gBaebbnrfifHhDYfgasaacH8akY=wiFfYdH8Gipec8Eeeu0xXdbba9frFj0=OqFfea0dXdd9vqai=hGuQ8kuc9pgc9s8qqaq=dirpe0xb9q8qiLsFr0=vr0=vr0dc8meaabaqaciaacaGaaeqabaqabeGadaaakeaacqWGsbGudaWgaaWcbaGaeGymaedabeaakiabcIcaOiabdsha0jabcMcaPiabg2da9iabdkfasnaaBaaaleaacqaIXaqmaeqaaOGaeiikaGIaeGimaaJaeiykaKIaemyzau2aaWbaaSqabeaacqGHsislcqWGRbWAdaWgaaadbaGaeGymaeJaeGinaqdabeaaliabdsha0baakiabgUcaRiabdUgaRnaaBaaaleaacqaIXaqmcqaIZaWmaeqaaOWaa8qmaeaadaWcaaqaaiabdkhaYbqaaiabdkhaYjabgUcaRiabdseaenaaBaaaleaacqaIYaGmaeqaaOGaeiikaGccciGae8hXdqNaeiykaKcaaaWcbaGaeGimaadabaGaemiDaqhaniabgUIiYdGccqWGLbqzdaahaaWcbeqaaiabdUgaRnaaBaaameaacqaIXaqmcqaI0aanaeqaaSGaeiikaGIae8hXdqNaeyOeI0IaemiDaqNaeiykaKcaaOGaemizaqMae8hXdqNaeiOla4caaa@60BC@

The central step of our proof is showing that R˜1
 MathType@MTEF@5@5@+=feaafiart1ev1aaatCvAUfKttLearuWrP9MDH5MBPbIqV92AaeXatLxBI9gBaebbnrfifHhDYfgasaacH8akY=wiFfYdH8Gipec8Eeeu0xXdbba9frFj0=OqFfea0dXdd9vqai=hGuQ8kuc9pgc9s8qqaq=dirpe0xb9q8qiLsFr0=vr0=vr0dc8meaabaqaciaacaGaaeqabaqabeGadaaakeaacuWGsbGugaacamaaBaaaleaacqaIXaqmaeqaaaaa@2F04@ and D˜2
 MathType@MTEF@5@5@+=feaafiart1ev1aaatCvAUfKttLearuWrP9MDH5MBPbIqV92AaeXatLxBI9gBaebbnrfifHhDYfgasaacH8akY=wiFfYdH8Gipec8Eeeu0xXdbba9frFj0=OqFfea0dXdd9vqai=hGuQ8kuc9pgc9s8qqaq=dirpe0xb9q8qiLsFr0=vr0=vr0dc8meaabaqaciaacaGaaeqabaqabeGadaaakeaacuWGebargaacamaaBaaaleaacqaIYaGmaeqaaaaa@2EEA@ are also related by (11). This will follow if we can show that for any differentiable function *f *= *f*(*τ*) and any fixed time interval [*s, t*]

lim⁡ε→0∫st(1−X1ε)(τ)f(τ) dτ=∫strr+D˜2(τ)f(τ)dτ.
 MathType@MTEF@5@5@+=feaafiart1ev1aaatCvAUfKttLearuWrP9MDH5MBPbIqV92AaeXatLxBI9gBaebbnrfifHhDYfgasaacH8akY=wiFfYdH8Gipec8Eeeu0xXdbba9frFj0=OqFfea0dXdd9vqai=hGuQ8kuc9pgc9s8qqaq=dirpe0xb9q8qiLsFr0=vr0=vr0dc8meaabaqaciaacaGaaeqabaqabeGadaaakeaadaWfqaqaaiGbcYgaSjabcMgaPjabc2gaTbWcbaacciGae8xTduMaeyOKH4QaeGimaadabeaakmaapedabaGaeiikaGIaeGymaeJaeyOeI0IaemiwaG1aaSbaaSqaaiabigdaXiab=v7aLbqabaGccqGGPaqkcqGGOaakcqWFepaDcqGGPaqkcqWGMbGzcqGGOaakcqWFepaDcqGGPaqkaSqaaiabdohaZbqaaiabdsha0bqdcqGHRiI8aOGaeeiiaaIaemizaqMae8hXdqNaeyypa0Zaa8qmaeaadaWcaaqaaiabdkhaYbqaaiabdkhaYjabgUcaRiqbdseaezaaiaWaaSbaaSqaaiabikdaYaqabaGccqGGOaakcqWFepaDcqGGPaqkaaaaleaacqWGZbWCaeaacqWG0baDa0Gaey4kIipakiabdAgaMjabcIcaOiab=r8a0jabcMcaPiabdsgaKjab=r8a0jabc6caUaaa@66A5@

To this end consider a partition {*s, s *+Δ,*s *+ 2Δ,..., *s *+ *n*Δ = *t*} of [*s, t*], where Δ = t−sn
 MathType@MTEF@5@5@+=feaafiart1ev1aaatCvAUfKttLearuWrP9MDH5MBPbIqV92AaeXatLxBI9gBaebbnrfifHhDYfgasaacH8akY=wiFfYdH8Gipec8Eeeu0xXdbba9frFj0=OqFfea0dXdd9vqai=hGuQ8kuc9pgc9s8qqaq=dirpe0xb9q8qiLsFr0=vr0=vr0dc8meaabaqaciaacaGaaeqabaqabeGadaaakeaadaWcaaqaaiabdsha0jabgkHiTiabdohaZbqaaiabd6gaUbaaaaa@31EE@. Then

∫st(1−X1ε)(τ)f(τ) dτ=∑k=0n−1∫s+kΔs+(k+1)Δ(1−X1ε)(τ)f(τ)dτ.
 MathType@MTEF@5@5@+=feaafiart1ev1aaatCvAUfKttLearuWrP9MDH5MBPbIqV92AaeXatLxBI9gBaebbnrfifHhDYfgasaacH8akY=wiFfYdH8Gipec8Eeeu0xXdbba9frFj0=OqFfea0dXdd9vqai=hGuQ8kuc9pgc9s8qqaq=dirpe0xb9q8qiLsFr0=vr0=vr0dc8meaabaqaciaacaGaaeqabaqabeGadaaakeaadaWdXaqaaiabcIcaOiabigdaXiabgkHiTiabdIfaynaaBaaaleaacqaIXaqmiiGacqWF1oqzaeqaaOGaeiykaKIaeiikaGIae8hXdqNaeiykaKIaemOzayMaeiikaGIae8hXdqNaeiykaKIaeeiiaacaleaacqWGZbWCaeaacqWG0baDa0Gaey4kIipakiabdsgaKjab=r8a0jabg2da9maaqahabaWaa8qmaeaacqGGOaakcqaIXaqmcqGHsislcqWGybawdaWgaaWcbaGaeGymaeJae8xTdugabeaakiabcMcaPiabcIcaOiab=r8a0jabcMcaPiabdAgaMjabcIcaOiab=r8a0jabcMcaPaWcbaGaem4CamNaey4kaSIaem4AaSMaeuiLdqeabaGaem4CamNaey4kaSIaeiikaGIaem4AaSMaey4kaSIaeGymaeJaeiykaKIaeuiLdqeaniabgUIiYdaaleaacqWGRbWAcqGH9aqpcqaIWaamaeaacqWGUbGBcqGHsislcqaIXaqma0GaeyyeIuoakiabdsgaKjab=r8a0jabc6caUaaa@72EA@

On [*s *+ *k*Δ, *s *+ (*k *+ 1)Δ] we have

*f*(*τ*) = *f*(*s *+ *k*Δ) + *O*(Δ)

so

∫s+kΔs+(k+1)Δ(1−X1ε)(τ)f(τ) dτ=[f(s+kΔ)+O(Δ)]∫s+kΔs+(k+1)Δ(1−X1ε)(τ) dτ
 MathType@MTEF@5@5@+=feaafiart1ev1aaatCvAUfKttLearuWrP9MDH5MBPbIqV92AaeXatLxBI9gBaebbnrfifHhDYfgasaacH8akY=wiFfYdH8Gipec8Eeeu0xXdbba9frFj0=OqFfea0dXdd9vqai=hGuQ8kuc9pgc9s8qqaq=dirpe0xb9q8qiLsFr0=vr0=vr0dc8meaabaqaciaacaGaaeqabaqabeGadaaakeaadaWdXaqaaiabcIcaOiabigdaXiabgkHiTiabdIfaynaaBaaaleaacqaIXaqmiiGacqWF1oqzaeqaaOGaeiykaKIaeiikaGIae8hXdqNaeiykaKIaemOzayMaeiikaGIae8hXdqNaeiykaKcaleaacqWGZbWCcqGHRaWkcqWGRbWAcqqHuoaraeaacqWGZbWCcqGHRaWkcqGGOaakcqWGRbWAcqGHRaWkcqaIXaqmcqGGPaqkcqqHuoara0Gaey4kIipakiabbccaGiabdsgaKjab=r8a0jabg2da9iabcUfaBjabdAgaMjabcIcaOiabdohaZjabgUcaRiabdUgaRjabfs5aejabcMcaPiabgUcaRiabd+eapjabcIcaOiabfs5aejabcMcaPiabc2faDnaapedabaGaeiikaGIaeGymaeJaeyOeI0IaemiwaG1aaSbaaSqaaiabigdaXiab=v7aLbqabaGccqGGPaqkcqGGOaakcqWFepaDcqGGPaqkaSqaaiabdohaZjabgUcaRiabdUgaRjabfs5aebqaaiabdohaZjabgUcaRiabcIcaOiabdUgaRjabgUcaRiabigdaXiabcMcaPiabfs5aebqdcqGHRiI8aOGaeeiiaaIaemizaqMae8hXdqhaaa@7FBD@

Because of the equicontinuity we have uniformly in *ε*

*D*_2,*ε*_(*τ*) = D˜2
 MathType@MTEF@5@5@+=feaafiart1ev1aaatCvAUfKttLearuWrP9MDH5MBPbIqV92AaeXatLxBI9gBaebbnrfifHhDYfgasaacH8akY=wiFfYdH8Gipec8Eeeu0xXdbba9frFj0=OqFfea0dXdd9vqai=hGuQ8kuc9pgc9s8qqaq=dirpe0xb9q8qiLsFr0=vr0=vr0dc8meaabaqaciaacaGaaeqabaqabeGadaaakeaacuWGebargaacamaaBaaaleaacqaIYaGmaeqaaaaa@2EEA@(*s *+ *k*Δ) + *O*(Δ) + *μ*(*ε*),

Where *μ*(*ε*) → 0 as *ε *→ 0 Hence, by the renewal reward result quoted earlier [15] we have almost surely

lim⁡ε→0∫s+kΔs+(k+1)Δ(1−X1ε)(τ)f(τ) dτ=rΔr+D˜2(s+kΔ)+O(Δ)f(s+kΔ)+O(Δ2)
 MathType@MTEF@5@5@+=feaafiart1ev1aaatCvAUfKttLearuWrP9MDH5MBPbIqV92AaeXatLxBI9gBaebbnrfifHhDYfgasaacH8akY=wiFfYdH8Gipec8Eeeu0xXdbba9frFj0=OqFfea0dXdd9vqai=hGuQ8kuc9pgc9s8qqaq=dirpe0xb9q8qiLsFr0=vr0=vr0dc8meaabaqaciaacaGaaeqabaqabeGadaaakeaadaWfqaqaaiGbcYgaSjabcMgaPjabc2gaTbWcbaacciGae8xTduMaeyOKH4QaeGimaadabeaakmaapedabaGaeiikaGIaeGymaeJaeyOeI0IaemiwaG1aaSbaaSqaaiabigdaXiab=v7aLbqabaGccqGGPaqkcqGGOaakcqWFepaDcqGGPaqkcqWGMbGzcqGGOaakcqaHepaDcqGGPaqkcqqGGaaiaSqaaiabdohaZjabgUcaRiabdUgaRjabfs5aebqaaiabdohaZjabgUcaRiabcIcaOiabdUgaRjabgUcaRiabigdaXiabcMcaPiabfs5aebqdcqGHRiI8aOGaemizaqMae8hXdqNaeyypa0ZaaSaaaeaacqWGYbGCcqqHuoaraeaacqWGYbGCcqGHRaWkcuWGebargaacamaaBaaaleaacqaIYaGmaeqaaOGaeiikaGIaem4CamNaey4kaSIaem4AaSMaeuiLdqKaeiykaKIaey4kaSIaem4ta8KaeiikaGIaeuiLdqKaeiykaKcaaiabdAgaMjabcIcaOiabdohaZjabgUcaRiabdUgaRjabfs5aejabcMcaPiabgUcaRiabd+eapjabcIcaOiabfs5aenaaCaaaleqabaGaeGOmaidaaOGaeiykaKcaaa@7BEF@

so

lim⁡ε→0∫st(1−X1ε)(τ)f(τ) dτ=∑k=0n−1rr+D˜2(s+kΔ)Δf(s+kΔ)+O(Δ),
 MathType@MTEF@5@5@+=feaafiart1ev1aaatCvAUfKttLearuWrP9MDH5MBPbIqV92AaeXatLxBI9gBaebbnrfifHhDYfgasaacH8akY=wiFfYdH8Gipec8Eeeu0xXdbba9frFj0=OqFfea0dXdd9vqai=hGuQ8kuc9pgc9s8qqaq=dirpe0xb9q8qiLsFr0=vr0=vr0dc8meaabaqaciaacaGaaeqabaqabeGadaaakeaadaWfqaqaaiGbcYgaSjabcMgaPjabc2gaTbWcbaacciGae8xTduMaeyOKH4QaeGimaadabeaakmaapedabaGaeiikaGIaeGymaeJaeyOeI0IaemiwaG1aaSbaaSqaaiabigdaXiab=v7aLbqabaGccqGGPaqkcqGGOaakcqWFepaDcqGGPaqkcqWGMbGzcqGGOaakcqWFepaDcqGGPaqkcqqGGaaiaSqaaiabdohaZbqaaiabdsha0bqdcqGHRiI8aOGaemizaqMae8hXdqNaeyypa0ZaaabCaeaadaWcaaqaaiabdkhaYbqaaiabdkhaYjabgUcaRiqbdseaezaaiaWaaSbaaSqaaiabikdaYaqabaGccqGGOaakcqWGZbWCcqGHRaWkcqWGRbWAcqqHuoarcqGGPaqkaaaaleaacqWGRbWAcqGH9aqpcqaIWaamaeaacqWGUbGBcqGHsislcqaIXaqma0GaeyyeIuoakiabfs5aejabdAgaMjabcIcaOiabdohaZjabgUcaRiabdUgaRjabfs5aejabcMcaPiabgUcaRiabd+eapjabcIcaOiabfs5aejabcMcaPiabcYcaSaaa@7491@

and in the limit Δ → 0 the right hand side converges to

∫strr+D˜2(τ)f(τ) dτ.
 MathType@MTEF@5@5@+=feaafiart1ev1aaatCvAUfKttLearuWrP9MDH5MBPbIqV92AaeXatLxBI9gBaebbnrfifHhDYfgasaacH8akY=wiFfYdH8Gipec8Eeeu0xXdbba9frFj0=OqFfea0dXdd9vqai=hGuQ8kuc9pgc9s8qqaq=dirpe0xb9q8qiLsFr0=vr0=vr0dc8meaabaqaciaacaGaaeqabaqabeGadaaakeaadaWdXaqaamaalaaabaGaemOCaihabaGaemOCaiNaey4kaSIafmiraqKbaGaadaWgaaWcbaGaeGOmaidabeaakiabcIcaOGGaciab=r8a0jabcMcaPaaacqWGMbGzcqGGOaakcqWFepaDcqGGPaqkaSqaaiabdohaZbqaaiabdsha0bqdcqGHRiI8aOGaeeiiaaIaemizaqMae8hXdqNaeiOla4caaa@44D4@

**Step 3**. The argument in step 2 and similar (but simpler) reasonings for the other dependent variables show that the *R*_*i*_, *P*_*i *_and *D*_*i*_, *i *= 1, 2 and the R˜i
 MathType@MTEF@5@5@+=feaafiart1ev1aaatCvAUfKttLearuWrP9MDH5MBPbIqV92AaeXatLxBI9gBaebbnrfifHhDYfgasaacH8akY=wiFfYdH8Gipec8Eeeu0xXdbba9frFj0=OqFfea0dXdd9vqai=hGuQ8kuc9pgc9s8qqaq=dirpe0xb9q8qiLsFr0=vr0=vr0dc8meaabaqaciaacaGaaeqabaqabeGadaaakeaacuWGsbGugaacamaaBaaaleaacqWGPbqAaeqaaaaa@2F6F@, P˜i
 MathType@MTEF@5@5@+=feaafiart1ev1aaatCvAUfKttLearuWrP9MDH5MBPbIqV92AaeXatLxBI9gBaebbnrfifHhDYfgasaacH8akY=wiFfYdH8Gipec8Eeeu0xXdbba9frFj0=OqFfea0dXdd9vqai=hGuQ8kuc9pgc9s8qqaq=dirpe0xb9q8qiLsFr0=vr0=vr0dc8meaabaqaciaacaGaaeqabaqabeGadaaakeaacuWGqbaugaacamaaBaaaleaacqWGPbqAaeqaaaaa@2F6B@ and D˜i
 MathType@MTEF@5@5@+=feaafiart1ev1aaatCvAUfKttLearuWrP9MDH5MBPbIqV92AaeXatLxBI9gBaebbnrfifHhDYfgasaacH8akY=wiFfYdH8Gipec8Eeeu0xXdbba9frFj0=OqFfea0dXdd9vqai=hGuQ8kuc9pgc9s8qqaq=dirpe0xb9q8qiLsFr0=vr0=vr0dc8meaabaqaciaacaGaaeqabaqabeGadaaakeaacuWGebargaacamaaBaaaleaacqWGPbqAaeqaaaaa@2F53@ are both solutions of the same initial value problem. By unique solvability it follows that these solutions are identical, so for example R˜1
 MathType@MTEF@5@5@+=feaafiart1ev1aaatCvAUfKttLearuWrP9MDH5MBPbIqV92AaeXatLxBI9gBaebbnrfifHhDYfgasaacH8akY=wiFfYdH8Gipec8Eeeu0xXdbba9frFj0=OqFfea0dXdd9vqai=hGuQ8kuc9pgc9s8qqaq=dirpe0xb9q8qiLsFr0=vr0=vr0dc8meaabaqaciaacaGaaeqabaqabeGadaaakeaacuWGsbGugaacamaaBaaaleaacqaIXaqmaeqaaaaa@2F04@(*t*) = *R*_1_(*t*) for all *t*. This uniqueness also implies (by a standard argument) that the passage to a subsequence of the *ε*_*n *_made earlier is not necessary, but that in fact lim_*ε*→0 _*R*_*iε*_(*t*) = *R*_*i*_(*t*) and likewise for all other dependent variables.

This completes the proof.

**Remark**. This result is only a first step in a possible more complete analysis of the whole process. Specifically, we intend to study the partial differential equations governing the probabilities that the stochastic variables *R*_*i*_, *P*_*i*_, *D*_*i *_assume values in certain ranges, derive the deterministic model given earlier as a set of equations for the first moments of these variables, and proceed to study fluctuations. The nonlinear coupling in our equations makes this a challenging program.

## Numerical tests

Here we present some results of simulations performed with the XPPAUT package (see [16,17]). The chosen parameters are those from Table 1. Figure 1 shows the time course of the proteins *P*_1 _and *P*_3 _for the deterministic model, which oscillate with a period of about 3 hours but differ slightly in their periods. A slight circadian variation is seen; it is much more promiment in Figure 2, where the responses of the protein products of the fifth DNA site are shown; note the time lag of *D*_5 _with respect to *D*_13_.

In Figures 3 and 4 the same calculation was done for the stochastic model. This calculation used Gillespie's method [11], where the *ε *was chosen as 2.8 × 10^-5^*hrs*. The results are essentially identical to the ones for the time-averaged model.

As a control measure we performed some calculations with larger *ε*, for example *ε *= 2.8 × 10^-3 ^hrs and *ε *= 0.028 hrs. For the former case, especially, the results were close to the time-averaged simulations. For the latter case, deviations from the time-averaged simulations became noticable: the amplitude of the circadian oscillations in *D*_5 _fluctuated stochastically and their period decreased slightly.

Despite these more significant stochastic effects with larger *ε*, the integrity of the circadian period is remarkably robust in our model with respect to the choice of *ε*. We demonstrate this by computing Fourier power spectra of *D*_5 _time series generated by simulations with *ε *= 2.8 × 10^-5 ^and *ε *= 2.8 × 10^-2 ^(see Figures 5 and 6). The former was calculated from a time series of 7447 data points at intervals of 1 minute, representing 124.1 hours of real time. The latter was calculated from a time series of 9920 data points at intervals of 10 minutes, representing 1653.2 hours of real time. We chose to integrate for a longer time in the latter case because the circadian oscillations were less regular. The power spectrum is shown in decibels (decibels = 10 log_10_(power), where power = |*X*_*i*_|^2 ^for *X*_*i*_, the *i*^*th *^frequency component of the Fourier transform of the time series {*x*_*k*_}). The frequencies of the primary oscillators show up clearly in the power spectra at close to 8 and 9 cycles per day respectively, and the circadian oscillations are clearly overwhelmingly dominant at close to (but not exactly) 1 cycle per day in both cases. Even after 65 "days" with *ε *= 0.028, the stochastic oscillator remained in phase with the circadian period; the wave form appeared to persist indefinitely.

## Remarks on the frequencies of the primary oscillators

The fundamental idea of our model is that circadian oscillations can easily be achieved via coupling of faster oscillators. We now address the question of whether the primary oscillators could attain circadian periods without need for coupling within reasonable ranges of parameter values based on known biochemistry. To this end we investigated which (if any) intrinsic limitations there are on the periods of the primary oscillators introduced earlier. We first explored (randomly) variations of the growth parameters *k*_13_, *k*_15_, *k*_17_, etc., and the unbinding rates *r *and *s *to see how they would affect the periods of the time-averaged single primary oscillator

dR1dt=k13rr+D2−k14R1dP1dt=k15R1−k16P1−2k17P12+2k18D1dD1dt=k17P12−k18D1dR2dt=k13D1s+D1−k14R2dP2dt=k25R2−k16P2−2k27P22+2k28D2dD2dt=k27P22−k28D2
 MathType@MTEF@5@5@+=feaafiart1ev1aaatCvAUfKttLearuWrP9MDH5MBPbIqV92AaeXatLxBI9gBaebbnrfifHhDYfgasaacH8akY=wiFfYdH8Gipec8Eeeu0xXdbba9frFj0=OqFfea0dXdd9vqai=hGuQ8kuc9pgc9s8qqaq=dirpe0xb9q8qiLsFr0=vr0=vr0dc8meaabaqaciaacaGaaeqabaqabeGadaaakeaafaqadeGbbaaaaeaadaWcaaqaaiabdsgaKjabdkfasnaaBaaaleaacqaIXaqmaeqaaaGcbaGaemizaqMaemiDaqhaaiabg2da9iabdUgaRnaaBaaaleaacqaIXaqmcqaIZaWmaeqaaOWaaSaaaeaacqWGYbGCaeaacqWGYbGCcqGHRaWkcqWGebardaWgaaWcbaGaeGOmaidabeaaaaGccqGHsislcqWGRbWAdaWgaaWcbaGaeGymaeJaeGinaqdabeaakiabdkfasnaaBaaaleaacqaIXaqmaeqaaaGcbaWaaSaaaeaacqWGKbazcqWGqbaudaWgaaWcbaGaeGymaedabeaaaOqaaiabdsgaKjabdsha0baacqGH9aqpcqWGRbWAdaWgaaWcbaGaeGymaeJaeGynaudabeaakiabdkfasnaaBaaaleaacqaIXaqmaeqaaOGaeyOeI0Iaem4AaS2aaSbaaSqaaiabigdaXiabiAda2aqabaGccqWGqbaudaWgaaWcbaGaeGymaedabeaakiabgkHiTiabikdaYiabdUgaRnaaBaaaleaacqaIXaqmcqaI3aWnaeqaaOGaemiuaa1aa0baaSqaaiabigdaXaqaaiabikdaYaaakiabgUcaRiabikdaYiabdUgaRnaaBaaaleaacqaIXaqmcqaI4aaoaeqaaOGaemiraq0aaSbaaSqaaiabigdaXaqabaaakeaadaWcaaqaaiabdsgaKjabdseaenaaBaaaleaacqaIXaqmaeqaaaGcbaGaemizaqMaemiDaqhaaiabg2da9iabdUgaRnaaBaaaleaacqaIXaqmcqaI3aWnaeqaaOGaemiuaa1aa0baaSqaaiabigdaXaqaaiabikdaYaaakiabgkHiTiabdUgaRnaaBaaaleaacqaIXaqmcqaI4aaoaeqaaOGaemiraq0aaSbaaSqaaiabigdaXaqabaaakeaadaWcaaqaaiabdsgaKjabdkfasnaaBaaaleaacqaIYaGmaeqaaaGcbaGaemizaqMaemiDaqhaaiabg2da9iabdUgaRnaaBaaaleaacqaIXaqmcqaIZaWmaeqaaOWaaSaaaeaacqWGebardaWgaaWcbaGaeGymaedabeaaaOqaaiabdohaZjabgUcaRiabdseaenaaBaaaleaacqaIXaqmaeqaaaaakiabgkHiTiabdUgaRnaaBaaaleaacqaIXaqmcqaI0aanaeqaaOGaemOuai1aaSbaaSqaaiabikdaYaqabaaakeaadaWcaaqaaiabdsgaKjabdcfaqnaaBaaaleaacqaIYaGmaeqaaaGcbaGaemizaqMaemiDaqhaaiabg2da9iabdUgaRnaaBaaaleaacqaIYaGmcqaI1aqnaeqaaOGaemOuai1aaSbaaSqaaiabikdaYaqabaGccqGHsislcqWGRbWAdaWgaaWcbaGaeGymaeJaeGOnaydabeaakiabdcfaqnaaBaaaleaacqaIYaGmaeqaaOGaeyOeI0IaeGOmaiJaem4AaS2aaSbaaSqaaiabikdaYiabiEda3aqabaGccqWGqbaudaqhaaWcbaGaeGOmaidabaGaeGOmaidaaOGaey4kaSIaeGOmaiJaem4AaS2aaSbaaSqaaiabikdaYiabiIda4aqabaGccqWGebardaWgaaWcbaGaeGOmaidabeaaaOqaamaalaaabaGaemizaqMaemiraq0aaSbaaSqaaiabikdaYaqabaaakeaacqWGKbazcqWG0baDaaGaeyypa0Jaem4AaS2aaSbaaSqaaiabikdaYiabiEda3aqabaGccqWGqbaudaqhaaWcbaGaeGOmaidabaGaeGOmaidaaOGaeyOeI0Iaem4AaS2aaSbaaSqaaiabikdaYiabiIda4aqabaGccqWGebardaWgaaWcbaGaeGOmaidabeaaaaaaaa@CF05@

Initially we kept the decay parameters *k*_14_, *k*_16_, *k*_18 _fixed and just varied *k*_13_. This had a modest effect on the period; the longest which was observed was 3.5 hrs. Random experiments of this nature did not produce periods of circadian length.

For a systematic investigation of the dependence of the periods on the parameters, we then set *k*_25 _= *k*_15_, *k*_27 _= *k*_17_, *k*_28 _= *k*_18 _and linearized the system about its unique positive equilibrium (*R*_1*E*_, *P*_1*E*_, *D*_1*E*_, *R*_2*E*_, *P*_2*E*_, *D*_2*E*_). The linearization yields the 6-by-6 matrix

A=(−k140000−k13r(r+D2E)2k15−k16−2k17P1E2k1800002k17P1E−k1800000k13s(s+D1E)2−k1400000k15−k16−2k17P2E2k1800002k17P2E−k18)
 MathType@MTEF@5@5@+=feaafiart1ev1aaatCvAUfKttLearuWrP9MDH5MBPbIqV92AaeXatLxBI9gBaebbnrfifHhDYfgasaacH8akY=wiFfYdH8Gipec8Eeeu0xXdbba9frFj0=OqFfea0dXdd9vqai=hGuQ8kuc9pgc9s8qqaq=dirpe0xb9q8qiLsFr0=vr0=vr0dc8meaabaqaciaacaGaaeqabaqabeGadaaakeaacqWGbbqqcqGH9aqpdaqadaqaauaabeqagyaaaaaabaGaeyOeI0Iaem4AaS2aaSbaaSqaaiabigdaXiabisda0aqabaaakeaacqaIWaamaeaacqaIWaamaeaacqaIWaamaeaacqaIWaamaeaadaWcaaqaaiabgkHiTiabdUgaRnaaBaaaleaacqaIXaqmcqaIZaWmaeqaaOGaemOCaihabaGaeiikaGIaemOCaiNaey4kaSIaemiraq0aaSbaaSqaaiabikdaYiabdweafbqabaGccqGGPaqkdaahaaWcbeqaaiabikdaYaaaaaaakeaacqWGRbWAdaWgaaWcbaGaeGymaeJaeGynaudabeaaaOqaaiabgkHiTiabdUgaRnaaBaaaleaacqaIXaqmcqaI2aGnaeqaaOGaeyOeI0IaeGOmaiJaem4AaS2aaSbaaSqaaiabigdaXiabiEda3aqabaGccqWGqbaudaWgaaWcbaGaeGymaeJaemyraueabeaaaOqaaiabikdaYiabdUgaRnaaBaaaleaacqaIXaqmcqaI4aaoaeqaaaGcbaGaeGimaadabaGaeGimaadabaGaeGimaadabaGaeGimaadabaGaeGOmaiJaem4AaS2aaSbaaSqaaiabigdaXiabiEda3aqabaGccqWGqbaudaWgaaWcbaGaeGymaeJaemyraueabeaaaOqaaiabgkHiTiabdUgaRnaaBaaaleaacqaIXaqmcqaI4aaoaeqaaaGcbaGaeGimaadabaGaeGimaadabaGaeGimaadabaGaeGimaadabaGaeGimaadabaWaaSaaaeaacqWGRbWAdaWgaaWcbaGaeGymaeJaeG4mamdabeaakiabdohaZbqaaiabcIcaOiabdohaZjabgUcaRiabdseaenaaBaaaleaacqaIXaqmcqWGfbqraeqaaOGaeiykaKYaaWbaaSqabeaacqaIYaGmaaaaaaGcbaGaeyOeI0Iaem4AaS2aaSbaaSqaaiabigdaXiabisda0aqabaaakeaacqaIWaamaeaacqaIWaamaeaacqaIWaamaeaacqaIWaamaeaacqaIWaamaeaacqWGRbWAdaWgaaWcbaGaeGymaeJaeGynaudabeaaaOqaaiabgkHiTiabdUgaRnaaBaaaleaacqaIXaqmcqaI2aGnaeqaaOGaeyOeI0IaeGOmaiJaem4AaS2aaSbaaSqaaiabigdaXiabiEda3aqabaGccqWGqbaudaWgaaWcbaGaeGOmaiJaemyraueabeaaaOqaaiabikdaYiabdUgaRnaaBaaaleaacqaIXaqmcqaI4aaoaeqaaaGcbaGaeGimaadabaGaeGimaadabaGaeGimaadabaGaeGimaadabaGaeGOmaiJaem4AaS2aaSbaaSqaaiabigdaXiabiEda3aqabaGccqWGqbaudaWgaaWcbaGaeGOmaiJaemyraueabeaaaOqaaiabgkHiTiabdUgaRnaaBaaaleaacqaIXaqmcqaI4aaoaeqaaaaaaOGaayjkaiaawMcaaaaa@AC57@

Its eigenvalues satisfy det (*A *- *λI*) = 0. This yields the characteristic equation

(*k*_14 _+ *λ*)^2 ^(*k*_18 _+ *λ*)^2 ^(*k*_16 _+ 2*k*_17_*P*_1*E *_+*λ*) (*k*_16 _+ 2*k*_17_*P*_2*E *_+*λ*) + c02
 MathType@MTEF@5@5@+=feaafiart1ev1aaatCvAUfKttLearuWrP9MDH5MBPbIqV92AaeXatLxBI9gBaebbnrfifHhDYfgasaacH8akY=wiFfYdH8Gipec8Eeeu0xXdbba9frFj0=OqFfea0dXdd9vqai=hGuQ8kuc9pgc9s8qqaq=dirpe0xb9q8qiLsFr0=vr0=vr0dc8meaabaqaciaacaGaaeqabaqabeGadaaakeaacqWGJbWydaqhaaWcbaGaeGimaadabaGaeGOmaidaaaaa@3008@ = 0.     (14)

Here,

c02=4k152k172k132rs(s+D1E)2(r+D2E)2.
 MathType@MTEF@5@5@+=feaafiart1ev1aaatCvAUfKttLearuWrP9MDH5MBPbIqV92AaeXatLxBI9gBaebbnrfifHhDYfgasaacH8akY=wiFfYdH8Gipec8Eeeu0xXdbba9frFj0=OqFfea0dXdd9vqai=hGuQ8kuc9pgc9s8qqaq=dirpe0xb9q8qiLsFr0=vr0=vr0dc8meaabaqaciaacaGaaeqabaqabeGadaaakeaacqWGJbWydaqhaaWcbaGaeGimaadabaGaeGOmaidaaOGaeyypa0ZaaSaaaeaacqaI0aancqWGRbWAdaqhaaWcbaGaeGymaeJaeGynaudabaGaeGOmaidaaOGaem4AaS2aa0baaSqaaiabigdaXiabiEda3aqaaiabikdaYaaakiabdUgaRnaaDaaaleaacqaIXaqmcqaIZaWmaeaacqaIYaGmaaGccqWGYbGCcqWGZbWCaeaacqGGOaakcqWGZbWCcqGHRaWkcqWGebardaWgaaWcbaGaeGymaeJaemyraueabeaakiabcMcaPmaaCaaaleqabaGaeGOmaidaaOGaeiikaGIaemOCaiNaey4kaSIaemiraq0aaSbaaSqaaiabikdaYiabdweafbqabaGccqGGPaqkdaahaaWcbeqaaiabikdaYaaaaaGccqGGUaGlaaa@541A@

To identify solutions with longer periods we look for a pair of eigenvalues with positive real part and small imaginary parts. Observe that c02
 MathType@MTEF@5@5@+=feaafiart1ev1aaatCvAUfKttLearuWrP9MDH5MBPbIqV92AaeXatLxBI9gBaebbnrfifHhDYfgasaacH8akY=wiFfYdH8Gipec8Eeeu0xXdbba9frFj0=OqFfea0dXdd9vqai=hGuQ8kuc9pgc9s8qqaq=dirpe0xb9q8qiLsFr0=vr0=vr0dc8meaabaqaciaacaGaaeqabaqabeGadaaakeaacqWGJbWydaqhaaWcbaGaeGimaadabaGaeGOmaidaaaaa@3008@ = 0 in (14) produces 6 real and negative eigenvalues (eigenvalues are counted with their multiplicity). If we now increase c02
 MathType@MTEF@5@5@+=feaafiart1ev1aaatCvAUfKttLearuWrP9MDH5MBPbIqV92AaeXatLxBI9gBaebbnrfifHhDYfgasaacH8akY=wiFfYdH8Gipec8Eeeu0xXdbba9frFj0=OqFfea0dXdd9vqai=hGuQ8kuc9pgc9s8qqaq=dirpe0xb9q8qiLsFr0=vr0=vr0dc8meaabaqaciaacaGaaeqabaqabeGadaaakeaacqWGJbWydaqhaaWcbaGaeGimaadabaGaeGOmaidaaaaa@3008@, one pair of eigenvalues approaches and eventually crosses the imaginary axis (Hopf bifurcation), producing the oscillations. However, the only way to force the crossing of the imaginary axis at small imaginary value is to move a pair of eigenvalues closer to the imaginary axis to begin with (i.e., when c02
 MathType@MTEF@5@5@+=feaafiart1ev1aaatCvAUfKttLearuWrP9MDH5MBPbIqV92AaeXatLxBI9gBaebbnrfifHhDYfgasaacH8akY=wiFfYdH8Gipec8Eeeu0xXdbba9frFj0=OqFfea0dXdd9vqai=hGuQ8kuc9pgc9s8qqaq=dirpe0xb9q8qiLsFr0=vr0=vr0dc8meaabaqaciaacaGaaeqabaqabeGadaaakeaacqWGJbWydaqhaaWcbaGaeGimaadabaGaeGOmaidaaaaa@3008@ = 0).

To achieve this, we first modified the parameter *k*_17 _governing the rate of homodimer formation. However, decreasing *k*_17 _turns out to increase *P*_1*E*_, counter-acting attempts to move the crossing pair closer to the real axis.

Finally, the actual rate constant of homodimer decay, *k*_18_, is not known, although it is unlikely to be smaller than 1 per hour. Choosing it to be exactly 1 per hour (earlier it was set to 15 per hour) we increased the periods up to 9 hours. Setting *k*_18 _this low is probably not reasonable, but given no *a priori *firm bounds as to how small *k*_18 _can actually be (a comment that applies to *k*_14 _and *k*_16 _as well), no simple predictions on the size of the periods of the primary oscillators can be made.

The following set of parameters produces a wavelength of about 22 hours:

*k*_13 _= 1000, *k*_14 _= *k*_16 _= 1, *k*_15 _= 400, *k*_17 _= 10^-5^, *k*_18 _= 0.25, *r *= 1, *s *= 9000. Thus almost circadian periods can be obtained, but only by stretching parameters beyond biochemically reasonable values.

## Conclusion

We have shown that TTOs in both their stochastic and time-averaged versions produce stable ultradian oscillations for reasonable parameter choices. Although the effect of the stochasticity is to degrade the circadian rhythms as in other models like that of Forger and Peskin [9], these oscillations are nevertheless robust in our model with respect to the scaling parameter governing the dimer-driven stochastic activation or inhibition of the relevant gene sites. Couplings of such TTOs with slight variations in their periods offer a simple mechanism to explain the emergence of circadian rhythms as "beats". This explanation has the added desirable feature of making circadian rhythms readily adaptable to evolutionary pressures.

## Competing interests

The author(s) declare that they have no competing interests.

## Authors' contributions

The model equations were developed by RI and RE based on information about the biochemical processes provided by VP. The biochemistry background and determination of parameters from the literature was contributed by VP. The implementation of the model and numerical simulations were done by RG and the mathematics by RI, RE and RG.
